# Induction of protective immunity in swine by recombinant bamboo mosaic virus expressing foot-and-mouth disease virus epitopes

**DOI:** 10.1186/1472-6750-7-62

**Published:** 2007-09-27

**Authors:** Chung-Da Yang, Jia-Teh Liao, Chen-Yen Lai, Ming-Hwa Jong, Chi-Ming Liang, Yeou-Liang Lin, Na-Sheng Lin, Yau-Heiu Hsu, Shu-Mei Liang

**Affiliations:** 1 Agricultural Biotechnology Research Center, Academia Sinica, Taipei 11529, Taiwan; 2 Institute of Plant and Microbial Biology, Academia Sinica, Taipei, Taiwan; 3 Graduate Institute of Biotechnology, National Chung Hsing University, Taichung, Taiwan; 4 National Institute for Animal Health, Taipei, Taiwan; 5 National Health Research Institutes, Zhunan, Taiwan

## Abstract

**Background:**

Plant viruses can be employed as versatile vectors for the production of vaccines by expressing immunogenic epitopes on the surface of chimeric viral particles. Although several viruses, including tobacco mosaic virus, potato virus X and cowpea mosaic virus, have been developed as vectors, we aimed to develop a new viral vaccine delivery system, a bamboo mosaic virus (BaMV), that would carry larger transgene loads, and generate better immunity in the target animals with fewer adverse environmental effects.

**Methods:**

We engineered the BaMV as a vaccine vector expressing the antigenic epitope(s) of the capsid protein VP1 of foot-and-mouth disease virus (FMDV). The recombinant BaMV plasmid (pBVP1) was constructed by replacing DNA encoding the 35 N-terminal amino acid residues of the BaMV coat protein with that encoding 37 amino acid residues (T^128^-N^164^) of FMDV VP1.

**Results:**

The pBVP1 was able to infect host plants and to generate a chimeric virion BVP1 expressing VP1 epitopes in its coat protein. Inoculation of swine with BVP1 virions resulted in the production of anti-FMDV neutralizing antibodies. Real-time PCR analysis of peripheral blood mononuclear cells from the BVP1-immunized swine revealed that they produced VP1-specific IFN-γ. Furthermore, all BVP1-immunized swine were protected against FMDV challenge.

**Conclusion:**

Chimeric BaMV virions that express partial sequence of FMDV VP1 can effectively induce not only humoral and cell-mediated immune responses but also full protection against FMDV in target animals. This BaMV-based vector technology may be applied to other vaccines that require correct expression of antigens on chimeric viral particles.

## Background

Foot-and-mouth disease virus (FMDV) is the etiological agent of foot-and-mouth disease (FMD) that infects cloven-hoofed animals such as pigs, sheep and cattle and causes serious damage in the livestock industry [1]. Although conventional vaccines based on the chemically inactivated virus are effective against FMDV [2], outbreaks of FMD sometimes result from virus escaping from vaccine production units or from the use of improperly inactivated virus [2-4]. Alternative approaches to produce an effective and safe FMD vaccine are needed to replace inactivated virus-based vaccines.

FMDV particles are composed of 60 copies of each of four capsid proteins termed VP1, VP2, VP3 and VP4, which are cleavage products of the capsid precursor polypeptide P1[5,6]. VP1, VP2 and VP3 form the outer capsid shell, whereas VP4 lines the interior surface [7]. Among these capsid proteins, VP1 contains the major antigenic domains of the virus [8-11], with its G-H loop including at its apex a highly conserved Arg-Gly-Asp (RGD) tripeptide, which can bind to integrins and facilitate the internalization of FMDV into target cells [12,13]. Therefore, many investigators have used VP1 as a candidate vaccine against FMDV [10].

Chimeric plant virus-derived vaccines against FMD have been described and used in experimental or natural hosts [14-16]. Cowpea mosaic virus (CPMV) expressing VP1 epitopes on the surface of the virus was first reported to react with FMDV-specific antiserum [14]. Tobacco mosaic virus (TMV) expressing the full VP1 protein or an epitope of VP1 was subsequently shown to induce protective immunity against FMDV in both mice and swine [15,16]. Although preliminary protection against FMDV in swine was demonstrated with TMV expressing the VP1 epitope [16], effort is needed to improve not only the replication efficiency and stability of such chimeric viruses but also the immune responses they induce [17].

Recently, potato virus X (PVX), a member of the *Potexvirus *genus, was reported to be an effective epitope presentation system for chimeric virus particle production [18-21]. Analysis by fiber diffraction pattern has shown that the surface features of PVX are more flexible than those of TMV [22], which likely contributes significantly to the accommodation of foreign peptides on the surface of the virus. Bamboo mosaic virus (BaMV) is also a flexuous rod-shaped member of the *Potexvirus *genus. It infects both monocotyledonous and dicotyledonous plants [23]. The viral genome of BaMV consists of a single-stranded positive-sense RNA molecule with a 5' cap structure and 3' poly(A) tail that contains five major open reading frames (ORFs) encoding different target proteins for viral replication, movement and assembly [24-27]. The ORF5 encodes a coat protein (CP) for virus encapsidation, cell-to-cell and long-distance movement [24].

Here we describe generation of a recombinant BaMV-based vector, namely pBVP1, by replacing complementary DNA (cDNA) encoding 35 amino acid residues from the N-terminal sequence of BaMV CP with cDNA encoding 37 amino acid residues (T^128^-N^164^) of FMDV (O/Taiwan/97) VP1. We examined the ability of this recombinant viral vector to generate a chimeric BaMV virus in plants and the effectiveness of the chimeric virus to induce immune responses and protection of swine against FMDV challenge.

## Methods

### Construction of an recombinant infectious pBVP1 vector

**Figure 1:**
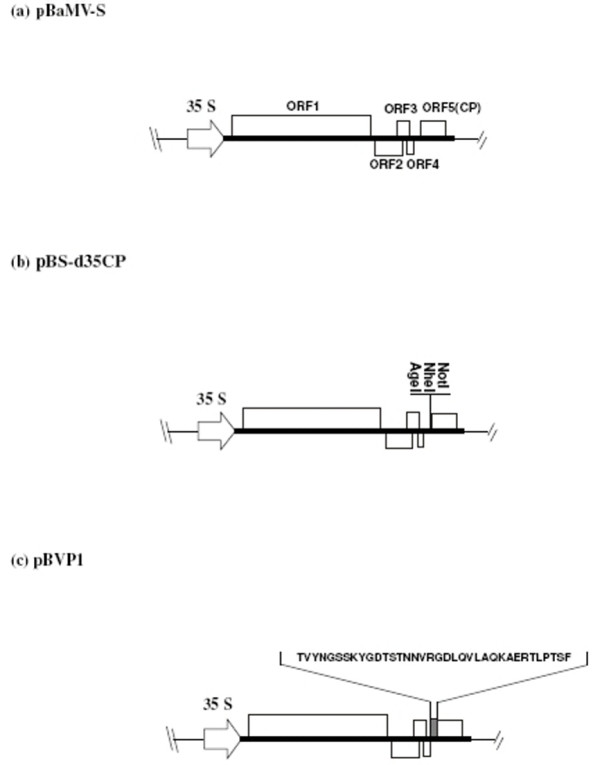
**Schematic representation of plasmids of wild-type BaMV-S as well as recombinant pBS-d35CP and pBVP1**. (a) pBaMV-S is an infectious BaMV cDNA plasmid under the control of cauliflower mosaic virus 35S promoter. The BaMV genome comprises 5 open reading frames (ORFs). The ORF1 encodes for viral replicase, whereas ORF2, ORF3 and ORF4 are called a "Triple-gene-block" that encodes viral movement proteins. ORF5 (CP) encodes a structural protein for virion formation. (b) pBS-d35CP is a mutated BaMV plasmid whose DNA coding for the N-terminal 35 amino acids of CP has been truncated, and multiple cloning sites have been engineered. (c) pBVP1 is a recombinant plasmid derived from pBS-d35CP by inserting the DNA coding for the 37 amino acid VP1 peptide to replace that coding for the N-terminal 35 amino acids of CP.

The full-length infectious cDNA of BaMV-S with an upstream cauliflower mosaic virus 35S promoter sequence was cloned in the plasmid pUC119 (Fig. 1a) as described previously [27]. A vector pBS-d35CP was derived from the aforementioned pBaMV-S plasmid by deletion of the N-terminal 35 amino acid sequence of CP and insertion of multiple cloning sites (*Age*I-*Nhe*I-*Not*I) by PCR (Fig. 1b). A sequence corresponding to amino acids 128–164 of VP1 of FMDV serotype O/Taiwan/97 was inserted into pBS-d35CP by PCR with the plasmid pVP1/Q15 used as a template [28]. The primers used were pr128164N (5'-GGgctagcAccatggACACCGTCTACAACGGGAG-3'; the sequence in small letters represents the sequentially ordered *Nhe*I and *Nco*I sites; the *Nco*I recognition sequence provided an AUG initiation codon) and pr128164C (5'-TTgcggccgcGTTGAAGGAGGTAGGC-3'; the sequence in small letters represents a *Not*I site). PCR was carried out at an initial temperature of 94°C for 5 min followed by 25 cycles of 94°C for 30 s, 50°C for 30 s and an extension at 72°C for 30 s. The amplified fragment encoding VP1 peptide was purified, sequenced and cloned into plasmid pBS-d35CP at the *Nhe*I and *Not*I sites. The sequence of the new plasmid was confirmed and denoted as pBVP1 (Fig. 1c).

### Virus propagation and purification

Plants of the local lesion host *Chenopodium quinoa *(*C. quinoa*) and systemic host *Nicotiana benthamiana *(*N. benthamiana*) were grown in a greenhouse exposed to normal daylight. For the infectivity assay, approximately 1 μg of purified viral DNA plasmids in 10 μl of double-distilled H_2_O was used to inoculate each leaf of test plants at the 6-leaf stage [27]. Observations for local lesions took place 10 days post-inoculation. For antigen preparation, 8 to 10 fully expanded leaves of *C. quinoa *plants that had been inoculated with pBaMV-S or pBVP1 were harvested at 10 days post-inoculation. The virions were subsequently purified from the leaves and the yield was determined by ultraviolet absorption as described previously [29]. The amount of VP1 epitope expressed in the chimeric virus BVP1 was estimated at about 14.3% of the total viral coat proteins. Purified virions were dissolved in BE buffer (10 mM Borate, pH 9.0, 1 mM EDTA), then stored at -20°C for subsequent immunization of swine.

### Western blotting analysis and immunogold labeling of virus particles

Rabbit anti-FMDV VP1 serum was prepared as described previously [28]. Total proteins were prepared from mock or viral DNA plasmid-inoculated *C. quinoa *with use of 1:2 (w/v) extraction buffer (50 mM Tris-HCl, pH 8.0, 10 mM KCl, 10 mM MgCl_2_, 1 mM EDTA, 20% glycerol and 2% SDS) and heated at 100°C for 5 min. Protein samples were separated by 12% SDS-PAGE and electrophoretically transferred to Immobilon-P membranes (Bio-Rad) with 200 mA for 1 h at 4°C. After blocking, the proteins on the membranes were probed with anti-BaMV-S CP or anti-FMDV VP1 antibodies or serum from FMDV-infected swine and then processed as described previously [27].

For immunogold labeling of virus particles, the pBaMV-S or pBVP1-inoculated leaves of *C. quinoa *were harvested at 10 days post-inoculation and the labeling procedures were conducted as described previously [30]. Briefly, grids were first floated on leaf extract for 5 min followed by diluted 1:100 anti-BaMV-S CP or anti-FMDV VP1 antibodies and then decorated by gold-labeled goat anti-rabbit IgG complexes. The grids were then stained with 2% uranyl acetate and examined under a Philips CM100 electron microscope.

### Analysis of the purity of BVP1 virions on SDS-PAGE

The recombinant virus BVP1 was purified from infected leaves and electrophoresed on a 12.5% polyacrylamide gel containing 1% SDS. The gel was stained with PlusOne Silver Staining Kit (Amersham Bioscience, Sweden).

### Immunization and viral challenge in swine

Specific pathogen-free female or castrated male swine (2 months old, weighing approximately 25 kg) were obtained in Taiwan. In experiment 1, two groups of three pigs each were immunized respectively by intramuscular injection into the neck muscles beside the ears with 10 mg and 5 mg of BVP1 virions emulsified with Montanide ISA 206 (Seppic, France) at a 1:1 ratio (v/v). In addition, two pigs were immunized with wild-type virus BaMV-S emulsified with Montanide ISA 206 and another two with sterile PBS buffer as negative control groups. All animals boosted with the same amount six weeks later. Sera were collected for analysis from the immunized animals at days 0, 28, 42, 56, and 70. Four weeks after boost, all swine were challenged with 0.5 ml of 10^5.0 ^TCID_50 _of FMDV O/Taiwan/97 by subcutaneous injection into the right front heel bulb. The swine were monitored daily for FMD symptoms for 14 days. Signs of FMD symptoms included elevated body temperature above 40°C for 3 successive days, lameness, vesicular lesions on the snout, and coronary bands on the legs [28]. In experiment 2, three groups of three swine each were vaccinated with 5 mg, 1 mg, and 0.5 mg BVP1 respectively and boosted at day 28 according to the same regimen. As negative controls, two pigs were immunized with wild-type virus BaMV-S and another two with sterile PBS buffer. Seven days after boost (i.e., on day 35), swine were injected with 0.5 ml of 10^5.0 ^TCID_50 _of FMDV (O/Taiwan/97) as in experiment 1. The viral challenge experiments were carried out in high containment facilities.

### FMDV preparation for swine challenge

The FMDV used in challenge experiments was produced from a homogenized mixture of vesicular fluid and epithelium of a pig infected with FMDV O/Taiwan/97. The titer of the virus was titrated in BHK-21 cells in accordance with FMD World Reference Laboratory, Pirbright, UK.

### ELISA for serum titer analysis

ELISA was performed as described previously with minor modifications [31]. In brief, 96-well microtiter plates were coated with recombinant VP1 protein (1 μg/well) in 0.1 M carbonate/bicarbonate buffer, pH 9.4, overnight at 4°C. Plates were blocked with 5% skim milk in PBS and washed three times with PBS containing 0.1% Tween-20 (PBST). Samples of 1:50 diluted serum in serial dilution were added to wells (50 μl/well) and incubated for 1 h at 37°C. After three washings, a 1: 3000 diluted biotinylated goat anti-swine IgG antibody was added for 1 h at 37°C. The plates were subsequently washed, and streptavidin:peroxidase (1:3000 dilution) was added. After incubation for 1 h at room temperature, the plates were washed again in PBST. Enzyme substrate 3, 3', 5, 5'-tetramethylbenzidine (Sigma) was then added, and the colorigenic reaction was carried out at room temperature for 10 min. Finally, an equal volume of 1 N H_2_SO_4 _was added to stop the reaction, and the absorbance at 450 nm was measured by an ELISA reader. The titer was defined as the reciprocal of the dilution that resulted in an absorbance value 50% of total value obtained from subtracting maximum absorbance with background absorbance. The maximum absorbance is the absorbance at the plateau (around O.D. = 3.5 – 3.7) of the curve obtained by plotting the optical density versus serial dilution of sera of immunized swine in a semi-logarithmical manner.

### Assay for anti-FMDV NAs

The anti-FMDV NAs assays were carried out in 96 wells flat-bottomed plates using BHK-21 cells as described in the OIE manual [32]. In brief, sera from test animals were inactivated at 56°C for 30 min. Two-fold serial dilution of sera were performed in duplicate and 50 μl of each were added to the wells. Fifty μl of 100 TCID_50 _of FMDV (O/Taiwan/97) was then added to each well. The plate was vortexed for 1 min and incubated at 37°C for 90 min. BHK-21 cell suspension (1 × 10^5^cells) in EMEM containing 5% fetal bovine serum was subsequently added to each well and incubated for 48 h. The cells were then observed under a microscope for the cytopathic effect (CPE) of the virus as indicated by disruption of cell monolayer and change of shape from spindle to round. Titers were expressed as the final dilution of serum present in the serum/virus mixture where 50% of wells are protected.

### Preparation of total RNA

Peripheral blood mononuclear cells (PBMCs) isolated from test swine were seeded in triplicate in 6-well culture plates at 1 × 10^7 ^cells per well in 2 ml of DMEM culture medium supplemented with 10% FBS and 1% penicillin and streptomycin. After overnight culture, cells were incubated with 5 μg/ml recombinant VP1 (rVP1), prepared as described previously [28], or 1 μg/ml of phytohaemagglutinin (PHA; Sigma) for 6 h at 37°C in 5% CO_2_. Cells stimulated with PHA were used as positive controls. Following the incubation, cells were lysed in TRIzol™ reagent (Invitrogen), and total cellular RNA was isolated according to the manufacturer's instructions. The concentration of total cellular RNA was quantified by determination of optical density at 260 nm. Total cellular RNA was reverse transcribed into cDNA by SuperScript III™ reverse transcriptase (Invitrogen). The resulting cDNA was amplified by real-time PCR.

### Measurement of IFN-γ production by real-time PCR

Total IFN-γ mRNA was measured by real-time PCR by use of a SYBR Green system in a LightCycler instrument (Roche Applied Science). Samples were assayed in a final volume of 20 μl with use of the FastStart DNA Master SYBR Green I Kit (Roche), including heat-activatable *Taq *polymerase, plus 4 mM MgCl_2_, each primer (primer sequences were SW-IFN-γ (F4), 5'-GCTCTGGGAAACTGAATGACTTCG; and SW-IFN-γ (R4), 5'- GACTTCTCTTCCGCTTTCTTAGGTTAG) at 0.5 μM and 2 μl of cDNA was prepared as described above. Following polymerase activation (95°C for 10 min), 40 cycles were run with a 15 s denaturation at 95°C, 2 s annealing at 60°C, and 15 s extension at 72°C. The temperature transition rate was 20°C/s for all steps. The amount of PCR product was measured once every cycle immediately after the 72°C incubation (extension step) by detection of the fluorescence associated with the binding of SYBR Green I to the amplification product. Fluorescence curves were analyzed with use of LightCycler software, version 3.0 (RocheApplied Science). The primers were synthesized by Invitrogen Life Technologies. For each sample, the amount of IFN-γ was determined by comparing with a standard curve and normalized by using β-actin as the internal reference. All samples were processed in triplicate.

### Detection of antibodies against the non-structural protein of FMDV

Infected animals produce antibodies to both the structural and non-structural proteins of FMDV. Therefore, the presence of antibodies against only structural but not non-structural proteins can differentiate vaccinated animals from infected ones [33]. The occurrence of FMDV infection in swine was determined by measuring elicitation of antibodies against non-structural 3ABC proteins. Sera from all groups of swine were collected 8 and 14 days post challenge. The presence of antibodies against the non-structural protein 3ABC of FMDV in sera were measured with use of the Ceditest^®^FMDV-NS strip ELISA kit (Cedi Diagnostics BV) [33] according to the manufacturer's instructions.

## Results

### Characterization of a recombinant infectious pBVP1 vector

To use a BaMV-based vector to express VP1 epitopes of FMDV, we first constructed a modified BaMV vector, pBS-d35CP, derived from a pBaMV-S vector (Fig. 1a) by deleting 35 amino acids from the N-terminal CP and inserting multiple cloning sites (Fig. 1b). A cDNA sequence corresponding to the FMDV (O/Taiwan/97) VP1 128–164 amino acids was then cloned into the CP truncated region of pBS-d35CP to generate the pBVP1 vector (Fig. 1c). We revealed the infectivity of the recombinant viral vector in both the systemic host *N. benthamiana *and the local lesion host *C. quinoa*. In *N. benthamiana*, infection with pBVP1 produced milder mosaic symptoms than that with pBaMV-S, the full-length infectious cDNA of wild-type BaMV. In *C. quinoa*, chlorotic local lesions formed after pBVP1 inoculation were also distinct from those caused by pBaMV-S.

### Detection of VP1 epitopes in plants infected with pBVP1

**Figure 2:**
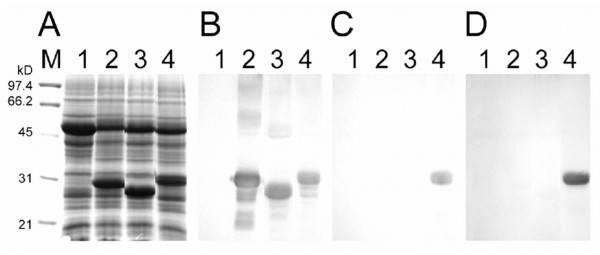
**Detection of VP1 antigenic epitopes in *Chenopodium quinoa *(*C. quinoa*) leaves inoculated with chimeric virus BVP1**. Leaves of *C. quinoa *were mock-inoculated (lane 1) or inoculated with wild-type pBaMV-S (lane 2), pBS-d35CP (lane 3) or pBVP1 (lane 4). Total proteins were prepared as described in Methods and electrophoretically separated on a 12% SDS-polyacrylamide gel, stained with Coomassie blue (panel A), or transferred to PVDF membrane, and detected with anti-BaMV-S CP serum (panel B), rabbit anti-FMDV VP1 serum (panel C), or serum from FMDV-infected swine (panel D).

We then determined whether pBVP1 could infect target plants to generate BaMV CP containing VP1 epitopes of FMDV. Total protein samples taken from *C. quinoa *leaves inoculated with distilled water (mock), pBaMV-S, pBS-d35CP or pBVP1 were respectively subjected to SDS-polyacrylamide gel electrophoresis (SDS-PAGE; Fig. 2A) and Western blotting analysis with use of anti-BaMV-S CP antibodies (Fig. 2B). As anticipated, protein extract of mock-inoculated leaves showed no BaMV CP (Fig. 2A and 2B, lane 1). The N-terminal truncated CP (Fig. 2A and 2B, lane 3), detected in the protein extract of pBS-d35CP-inoculated plants, migrated faster than the wild-type CP of pBaMV-S-infected plants (Fig. 2A and 2B, lane 2), which in turns migrated slightly faster than the chimeric CP from pBVP1 inoculated plants (Fig. 2A and 2B, lane 4). These results indicate an apparent size difference between the CP generated from pBVP1 and those from pBaMV-S and pBS-d35CP.

To further verify that CP generated from pBVP1-inoculated plants contained the VP1 antigenic epitopes of FMDV, Western blotting analysis with use of rabbit serum against FMDV VP1 (Fig. 2C) or serum from FMDV-infected swine (Fig. 2D) was undertaken. Both sera recognized a protein band of 31 kDa corresponding to the chimeric CP (Fig. 2C and 2D, lane 4). In contrast, no protein band was detected in the wild-type CP of BaMV-S (Fig. 2C and 2D, lane 2) or truncated CP of BS-d35CP (Fig. 2C and 2D, lane 3). Additional immunoblotting studies with the same antibodies in *C. quinoa *showed that even after five subsequent passages, protein extracts of pBVP1-inoculated leaves still contained a major protein band with similar mobility to chimeric CP (data not shown). Taken together, these results suggest that the inoculation of plants with pBVP1 plasmid generates a fusion protein comprised of truncated CP of BaMV and the VP1 epitopes of FMDV.

### Expression of FMDV VP1 epitopes on BVP1 virus particles

**Figure 3:**
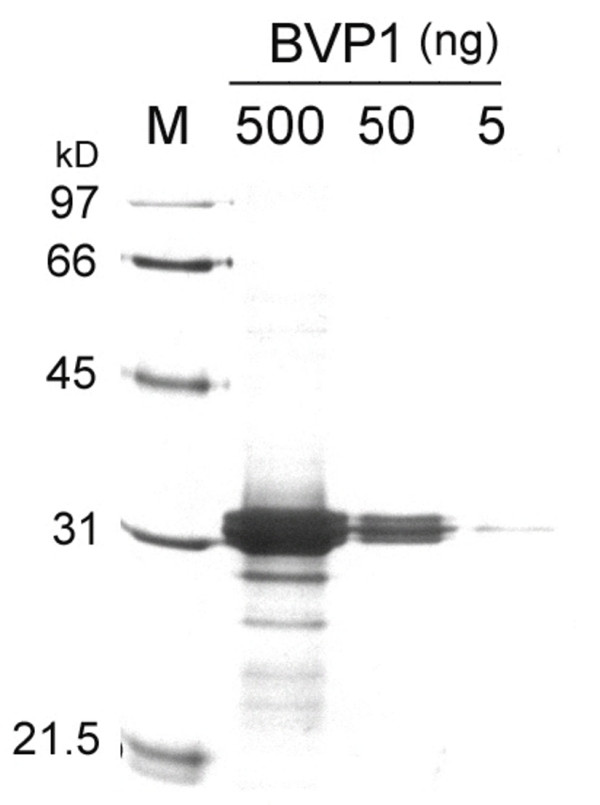
**Analysis of purified BVP1 virions on SDS- PAGE**. Various amounts of BVP1 virions purified from infected leaves by CsCl_2 _density gradient centrifugation were analyzed by electrophoresis through a 12.5% polyacrylamide and stained with PlusOne Silver Staining Kit (Amersham Bioscience, Sweden). Lane M, molecular weight standards. The relative molecular weights were as indicated on the left.

**Figure 4:**
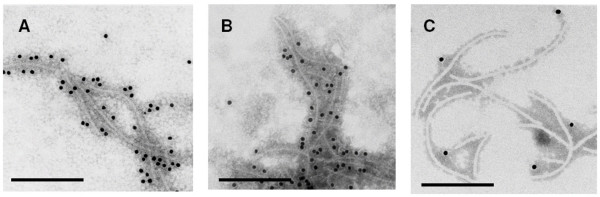
**Immunoelectron microscopy for identification of BaMV CP and FMDV VP1 on the surface of virus particles**. Leaf dips from *C. quinoa *infected with pBVP1 (A, B) or pBaMV-S (C) were obtained 10 days post-inoculation. Grids were first incubated with leaf extract and coated with diluted anti-BaMV CP serum (A) or anti-FMDV VP1 serum (B, C) followed by gold-labeled goat anti-rabbit IgG complexes. Grids were inspected in a Philips CM100 electron microscope. All bars represent 250 nm.

To further confirm that the FMDV VP1 epitopes was expressed on the viral surface, the chimeric virus, namely BVP1, was isolated from pBVP1-infected *C. quinoa *leaf tissue. The yield of the purified virus was estimated to be around 0.2–0.5 mg per gram of fresh leaf tissue. The purified BVP1 virus was analyzed by gel electrophoresis and silver staining. As shown in Fig. 3, small amounts of virus samples (5 ng and 50 ng) showed only single major band. When larger amount of virions (500 ng) was loaded, the majority of proteins migrated as 31 kD of chimeric CP while several low molecular weight bands of degraded CP were detected. The presence of the FMDV VP1 peptide on the virus surface was subsequently determined by using anti-BaMV CP or anti-FMDV VP1 antibodies, and the formation of the antibody-antigen complexes was detected with use of gold-labeled secondary antibodies and immunoelectron microscopy. We found that chimeric BVP1 virions were specifically labeled with anti-BaMV CP and anti-FMDV VP1 respectively (Fig. 4A and 4B). In contrast, BaMV-S virions were merely labeled with anti-BaMV CP serum (data not shown) but not with anti-FMDV VP1 (Fig. 4C). By negatively staining, no difference in morphology could be observed between wild type BaMV and chimeric virus BVP1. These data indicate that the FMDV VP1 peptide is fused to BaMV CP and expressed on the viral surface.

### Antibody responses elicited by chimeric virus BVP1 in swine

**Figure 5:**
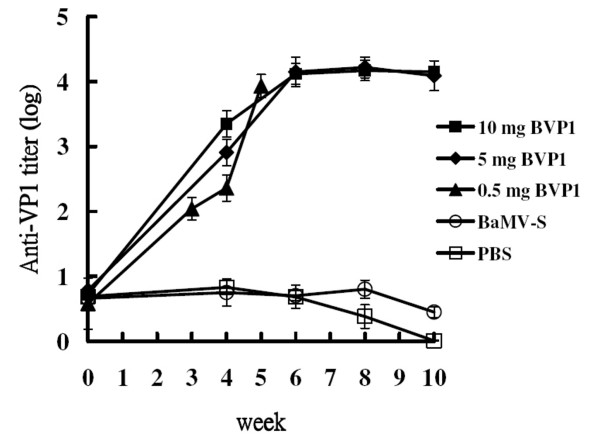
**Serum titers of swine immunized with chimeric virus BVP1. **Groups of swine were immunized with 0.5 mg (▲), 5 mg (◆), 10 mg (■) of chimeric virus BVP1 or 5 mg of wild-type virus BaMV-S (○) or PBS (□). Swine sera were collected at the indicated time after immunization. Anti-VP1 titers were determined by ELISA. The animals inoculated with 0.5 mg BVP1 were boosted with the same amount of BVP1 four weeks after priming while those inoculated with 5 mg and 10 mg of BVP1 were boosted six weeks after priming.

We then evaluated the ability of this chimeric virus to trigger immunity against FMDV in swine. In our first experiment, two groups of three pigs each were given intramuscular injections of 5 mg and 10 mg of BVP1 chimeric virus respectively and boosted with similar amount of BVP1 6 weeks later. Another two groups of two pigs each were vaccinated with 5 mg BaMV-S wild-type virus or PBS buffer respectively as negative controls. Sera obtained from both 5 mg and 10 mg BVP1-immunized swine elicited high levels of anti-VP1 antibodies, as measured by ELISA. In contrast, swine immunized with BaMV-S or PBS buffer all showed little, if any, anti-VP1 antibodies (Fig. 5). Furthermore, neutralizing antibodies (NAs) were detected in the sera of the BVP1-vaccinated groups but not in those of the negative control groups (Table 1). To confirm that even smaller amounts of BVP1 could exhibit similar effects, we immunized swine with either 0.5 mg or 1 mg of BVP1 and boosted with similar amounts of BVP1 4 weeks later. Substantial titers of anti-VP1 antibodies (Fig. 5) and NAs (Table 1) were detected even in the sera of swine given one inoculation of 0.5 mg BVP1, indicating that the chimeric BVP1 virus can effectively trigger specific NAs in swine.

**Table 1:** Titers of neutralizing antibodies (NAs) in swine immunized with chimeric virus BVP1

Experiment 1				
		NA titers ^a^		
		
Antigens	Swine No.	4W	6W	10W

10 mg BVP1	858	8	64	11
	859	11	362	362
	860	16	256	256

5 mg BVP1	861	6	32	23
	862	91	362	512
	863	64	256	256

5 mg BaMV-S	864	**–**	**–**	**–**
	865	**–**	**–**	**–**

PBS	866	**–**	**–**	**–**
	867	**–**	**–**	**–**

Experiment 2				

		NA titers ^a^		
		
Antigens	Swine No.	3W	4W	5W

5 mg BVP1	1	4	8	11
	2	32	128	128
	3	16	64	45

1 mg BVP1	4	16	32	256
	5	4	16	23
	6	32	32	32

0.5 mg BVP1	7	8	64	91
	8	**–**	8	32
	9	8	16	181

5 mg BaMV-S	10	**–**	**–**	**–**
	11	**–**	**–**	**–**

PBS	12	**–**	**–**	**–**
	13	**–**	**–**	**–**

^a ^The titer of NAs from each swine was measured at indicated weeks as described in Methods. (-) means that no NAs were detected at the highest concentration tested.

### IFN-γ production in immunized swine

To evaluate whether BVP1-immunized swine could induce a cell-mediated immune response in addition to NAs (a humoral response), we analyzed the ability of PBMCs to produce IFN-γ, a cytokine that plays an important role in cell-mediated immune responses. Real-time PCR analysis of specific IFN-γ mRNA showed that upon VP1 antigen stimulation, PBMCs from BVP1-immunized swine induced production of IFN-γ whereas BaMV-S or PBS-immunized swine did not (Table 2). As control, PBMCs from all groups of swine were stimulated with T-cell mitogen PHA (1 μg/ml) and found to produce similar amounts of IFN-γ (Table 2).

**Table 2:** IFN-γ production and protection in swine immunized with chimeric virus BVP1

		IFN-γ (fold change)^a^		
			
Antigens	Swine No.	rVP1	PHA	Clinical symptoms^b^
10 mg BVP1	858	3.8	2.4	No
	859	4.1	2.8	No
	860	3.4	2.2	No

5 mg BVP1	861	3.1	2.9	No
	862	3.8	2.1	No
	863	2.4	2.6	No

5 mg BaMV-S	864	1.0	2.5	Yes
	865	1.1	2.2	Yes

PBS	866	1.0	2.6	Yes
	867	1.0	2.4	Yes

^a ^PBMCs isolated from swine 10 weeks after priming were stimulated with 5 μg/ml of rVP1 or 1 μg/ml of PHA for 6 h at 37°C. Total cellular RNA was then extracted for the assay of IFN-γ production by real-time PCR. The fold change was determined by dividing the quantity of specific IFN-γ mRNA from stimulated cells by the quantity of IFN-γ mRNA from the unstimulated cells.

^b ^Swine of all groups were challenged with FMDV (O/Taiwan/97) and monitored daily for clinical symptoms of FMD for 14 days as described in Methods.

### Protection against FMDV challenge in immunized swine

We then determined whether BVP1 could confer immunoprotection in swine. All groups of swine were challenged with 10^5.0 ^TCID_50 _(50% tissue culture infective dose) of FMDV (O/Taiwan/97) and monitored for the appearance of FMD symptoms for two weeks. At days 2–4 after challenge, the negative control groups showed serious symptoms of FMD. In contrast, all swine immunized with BVP1 showed no FMD symptoms after challenge. In addition, since infected animals produce antibodies to both the structural and non-structural proteins of FMDV, the presence of antibodies against only structural but not non-structural proteins can differentiate vaccinated animals from infected ones [33]. We thus examined the presence of FMDV in sera of all animals 8–14 days after FMDV challenge by measuring antibodies against non-structural protein 3ABC. All the groups immunized with BVP1 did not develop an antibody response to 3ABC while control groups became 3ABC positive (Table 3).

**Table 3:** Detection of anti-3ABC antibodies and clinical symptoms in swine after FMDV challenge

Antigens	Swine No.	Anti-3ABC^a^	Clinical symptoms
5 mg BVP1	1	-	No
	2	-	No
	3	-	No

1 mg BVP1	4	-	No
	5	-	No
	6	-	No

0.5 mg BVP1	7	-	No
	8	-	No
	9	-	No

BaMV-S	10	+	Yes
	11	+	Yes

PBS	12	+	Yes
	13	+	Yes

^a ^The presence of antibodies against 3ABC of FMDV were measured 8 and 14 days post challenge, respectively. (+) indicates the presence of antibodies against 3ABC protein. (-) indicates no antibodies against the 3ABC protein.

## Discussion

Although use of CPMV and TMV as a vector to generate chimeric plant virus-derived vaccines against FMD has been described [14-16], effort is needed to improve not only the stability of such chimeric viruses but also the immune responses and protection they induce in the target animals [17]. Recently developed was a modified TMV-based vector that allows for the expression of peptides as long as 25 amino acids. Such chimeric constructs, however, are not as effective as the TMV expressing the 11 amino acid epitope in generating protective immunity [34]. In this study, we infected plants with plasmid pBVP1 to generate a chimeric virion (BVP1) expressing a 37 amino acid peptide of VP1 and elicited strong immunity in swine even after just one inoculation (Table 1 and Fig. 5). The BaMV-based expression system therefore may be better than the TMV system in expressing peptides up to 37 amino acids or longer. However, as different transgenes were used in BaMV, CPMV and TMV, it is difficult to judge at present whether chimeric BaMV is better than chimeric CPMV and TMV in terms of their elicitation of immune responses.

The prominent G-H loop of the capsid protein VP1 of FMDV, including at its apex a highly conserved Arg-Gly-Asp (RGD) tripeptide, has been identified as a major B-cell epitope for eliciting NAs [8-11]. However, administering G-H loop synthetic peptides alone in cattle and swine has resulted in limited induction of NAs or ineffective protection [35]. Piatti and others [36] showed induction of both T-cell immunity and high NA response positively correlated with effective protection in experimental animals immunized with inactivated FMDV. Parida and coauthors [37] have thus suggested that evaluation of effective protection of FMD vaccine should combine the presence of NAs and IFN-γ production (an indicator of cell-mediated immunity). Because the VP1 sequence (residues 135–160) of FMDV O1 Campos contains not only the G-H loop, the major B-cell epitope, but also the immunodominant T epitopes [38-40], in this study, we introduced residues 128–164, which includes T and B epitopes, of the VP1 sequence into the BaMV-based vector. Swine immunized with BVP1 elicited VP1-specific IFN-γ and high titers of NA against FMDV (Tables 1 and 2). To our knowledge, this is the first report of a chimeric plant virus correctly expressing VP1 epitope(s) to properly induce both humoral (as indicated by NAs) and cell-mediated immune responses (as indicated by VP1-specific IFN-γ production).

Most importantly, our results have clearly demonstrated that BVP1 immunization can protect swine against a challenge of 10^5.0 ^TCID_50 _FMDV, an amount 10 times higher than that recommended by the World Organization for Animal Health [32]. In addition, we have recently examined whether one inoculation is sufficient to protect the animals. Our preliminary results revealed that swine immunized once with 1 mg BVP1 were protected from FMDV challenge. We thus propose that BVP1 may be superior to other plant viruses such as TMV in presenting the VP1 peptide so that the immunogenic site(s) of the peptide mimic the conformation of the major antigenic epitope(s) of FMDV, thereby generating high titers of NAs as well as cellular immune responses. More studies involving the incorporation of the similar transgene into TMV and evaluation of the optimal dosage for protection, however, must be undertaken to confirm this proposal. Of note, an important advantage of the BaMV-based vector over CPMV, TMV and most other plant virus-based vectors is that BaMV is not a pathogen for a variety of crops and therefore may be ecologically safer for field use [23].

## Conclusion

We have inserted a transgene encoding a partial sequence of the capsid VP1 of FMDV in the plant virus BaMV. The recombinant plasmid could infect host plants to generate chimeric virions that elicit significant NA titers in the animals even after just one inoculation. The capability of this BaMV-based vector to carry larger transgenes, effectively express foreign peptide epitope(s) and induce protective humoral and cellular immune responses would be advantageous for its application in the development of vaccines against not only FMDV but also many other pathogens.

## Competing interests

As the content described in this paper has commercial potential, patent applications are being processed.

## Authors' contributions

CDY carried out the vaccine formulation, analyzed immune responses, and drafted the manuscript. CYL set up real-time PCR for IFN-γ analysis. JTL, YHH and NSL constructed BaMV-based vectors, purified chimeric BVP1 and performed immunoelectron microscopy. MHJ and YLL participated in swine immunization and challenge. CML and SML designed and coordinated the study and wrote the manuscript. All authors read and approved the final manuscript.

## References

[B1] Woolhouse M, Chase-Topping M, Haydon D, Friar J, Matthews L, Hughes G, Shaw D, Wilesmith J, Donaldson A, Cornell S, Keeling M, Grenfell B (2001). Epidemiology. Foot-and-mouth disease under control in the UK. Nature.

[B2] Barteling SJ, Vreeswijk J (1991). Developments in foot-and-mouth disease vaccines. Vaccine.

[B3] Brown F (1992). New approaches to vaccination against foot-and-mouth disease. Vaccine.

[B4] Doel TR (2003). FMD vaccines. Virus Res.

[B5] Belsham GJ (1993). Distinctive features of foot-and-mouth disease virus, a member of the picornavirus family; aspects of virus protein synthesis, protein processing and structure. Prog Biophys Mol Biol.

[B6] Saiz M, Nunez JI, Jimenez-Clavero MA, Baranowski E, Sobrino F (2002). Foot-and-mouth disease virus: biology and prospects for disease control. Microbes Infect.

[B7] Domingo E, Baranowski E, Escarmis C, Sobrino F (2002). Foot-and-mouth disease virus. Comp Immunol Microbiol Infect Dis.

[B8] Volpina OM, Surovoy AY, Zhmak MN, Kuprianova MA, Koroev DO, Chepurkin AV, Toloknov AS, Ivanov VT (1999). A peptide construct containing B-cell and T-cell epitopes from the foot-and-mouth disease viral VP1 protein induces efficient antiviral protection. Vaccine.

[B9] Wang CY, Chang TY, Walfield AM, Ye J, Shen M, Chen SP, Li MC, Lin YL, Jong MH, Yang PC, Chyr N, Kramer E, Brown F (2002). Effective synthetic peptide vaccine for foot-and-mouth disease in swine. Vaccine.

[B10] Grubman MJ, Baxt B (2004). Foot-and-mouth disease. Clin Microbiol Rev.

[B11] Oliveira E, Jimenez-Clavero MA, Nunez JI, Sobrino F, Andreu D (2005). Analysis of the immune response against mixotope peptide libraries from a main antigenic site of foot-and-mouth disease virus. Vaccine.

[B12] Baxt B, Morgan DO, Robertson BH, Timpone CA (1984). Epitopes on foot-and-mouth disease virus outer capsid protein VP1 involved in neutralization and cell attachment. J Virol.

[B13] Peng JM, Liang SM, Liang CM (2004). VP1 of foot-and-mouth disease virus induces apoptosis via the Akt signaling pathway. J Biol Chem.

[B14] Usha R, Rohll JB, Spall VE, Shanks M, Maule AJ, Johnson JE, Lomonossoff GP (1993). Expression of an animal virus antigenic site on the surface of a plant virus particle. Virology.

[B15] Wigdorovitz A, Perez Filgueira DM, Robertson N, Carrillo C, Sadir AM, Morris TJ, Borca MV (1999). Protection of mice against challenge with foot and mouth disease virus (FMDV) by immunization with foliar extracts from plants infected with recombinant tobacco mosaic virus expressing the FMDV structural protein VP1. Virology.

[B16] Wu L, Jiang L, Zhou Z, Fan J, Zhang Q, Zhu H, Han Q, Xu Z (2003). Expression of foot-and-mouth disease virus epitopes in tobacco by a tobacco mosaic virus-based vector. Vaccine.

[B17] Ahlquist P, Schwartz M, Chen J, Kushner D, Hao L, Dye BT (2005). Viral and host determinants of RNA virus vector replication and expression. Vaccine.

[B18] Brennan FR, Jones TD, Longstaff M, Chapman S, Bellaby T, Smith H, Xu F, Hamilton WD, Flock JI (1999). Immunogenicity of peptides derived from a fibronectin-binding protein of S. aureus expressed on two different plant viruses. Vaccine.

[B19] Marusic C, Rizza P, Lattanzi L, Mancini C, Spada M, Belardelli F, Benvenuto E, Capone I (2001). Chimeric plant virus particles as immunogens for inducing murine and human immune responses against human immunodeficiency virus type 1. J Virol.

[B20] Uhde K, Fischer R, Commandeur U (2005). Expression of multiple foreign epitopes presented as synthetic antigens on the surface of Potato virus X particles. Arch Virol.

[B21] Marconi G, Albertini E, Barone P, De Marchis F, Lico C, Marusic C, Rutili D, Veronesi F, Porceddu A (2006). In planta production of two peptides of the Classical Swine Fever Virus (CSFV) E2 glycoprotein fused to the coat protein of potato virus X. BMC Biotechnol.

[B22] Parker L, Kendall A, Stubbs G (2002). Surface features of potato virus X from fiber diffraction. Virology.

[B23] Hsu YH, Lin NS, Lapierre H, Signoret PA (2004). Bamboo mosaic. Viruses and Virus Disease of Poaceae, Gramineae.

[B24] Lin NS, Lin BY, Lo NW, Hu CC, Chow TY, Hsu YH (1994). Nucleotide sequence of the genomic RNA of bamboo mosaic potexvirus. J Gen Virol.

[B25] Yang CC, Liu JS, Lin CP, S. LN (1997). Nucleotide sequence and phylogenetic analysis of a bamboo mosaic potexvirus isolate from common bamboo (Bambusa vulgaris McClure). Bot Bull Acad Sin.

[B26] Li YI, Cheng YM, Huang YL, Tsai CH, Hsu YH, Meng M (1998). Identification and characterization of the Escherichia coli-expressed RNA-dependent RNA polymerase of bamboo mosaic virus. J Virol.

[B27] Lin MK, Chang BY, Liao JT, Lin NS, Hsu YH (2004). Arg-16 and Arg-21 in the N-terminal region of the triple-gene-block protein 1 of Bamboo mosaic virus are essential for virus movement. J Gen Virol.

[B28] Wang JH, Liang CM, Peng JM, Shieh JJ, Jong MH, Lin YL, Sieber M, Liang SM (2003). Induction of immunity in swine by purified recombinant VP1 of foot-and-mouth disease virus. Vaccine.

[B29] Lin NS, Chen CC (1991). Association of bamboo mosaic virus (BaMV) and BaMV-specific electron-dense crystalline bodies with chloroplasts.. Phytopathology.

[B30] Lin NS (1984). Gold-IgG complexes improve the detection and identification of viruses in leaf dip preparations. J Virol Methods.

[B31] Shieh JJ, Liang CM, Chen CY, Lee F, Jong MH, Lai SS, Liang SM (2001). Enhancement of the immunity to foot-and-mouth disease virus by DNA priming and protein boosting immunization. Vaccine.

[B32] OIE, Foot and mouth disease, in: Manual of Standards for Diagnostic Tests and Vaccines 2000, CHAPTER 2.1.1. 2002. http://www.oie.int/eng/normes/mmanual/a_00024.htm.

[B33] Sorensen KJ, Madsen KG, Madsen ES, Salt JS, Nqindi J, Mackay DK (1998). Differentiation of infection from vaccination in foot-and-mouth disease by the detection of antibodies to the non-structural proteins 3D, 3AB and 3ABC in ELISA using antigens expressed in baculovirus. Arch Virol.

[B34] Jiang L, Li Q, Li M, Zhou Z, Wu L, Fan J, Zhang Q, Zhu H, Xu Z (2006). A modified TMV-based vector facilitates the expression of longer foreign epitopes in tobacco. Vaccine.

[B35] Meloen RH, Casal JI, Dalsgaard K, Langeveld JP (1995). Synthetic peptide vaccines: success at last. Vaccine.

[B36] Piatti PG, Berinstein A, Lopez OJ, Borca MV, Fernandez F, Schudel AA, Sadir AM (1991). Comparison of the immune response elicited by infectious and inactivated foot-and-mouth disease virus in mice. J Gen Virol.

[B37] Parida S, Oh Y, Reid SM, Cox SJ, Statham RJ, Mahapatra M, Anderson J, Barnett PV, Charleston B, Paton DJ (2006). Interferon-gamma production in vitro from whole blood of foot-and-mouth disease virus (FMDV) vaccinated and infected cattle after incubation with inactivated FMDV. Vaccine.

[B38] Zamorano P, Wigdorovitz A, Chaher MT, Fernandez FM, Carrillo C, Marcovecchio FE, Sadir AM, Borca MV (1994). Recognition of B and T cell epitopes by cattle immunized with a synthetic peptide containing the major immunogenic site of VP1 FMDV 01 Campos. Virology.

[B39] Zamorano P, Wigdorovitz A, Perez-Filgueira M, Carrillo C, Escribano JM, Sadir AM, Borca MV (1995). A 10-amino-acid linear sequence of VP1 of foot and mouth disease virus containing B- and T-cell epitopes induces protection in mice. Virology.

[B40] Zamorano PI, Wigdorovitz A, Perez Filgueira DM, Escribano JM, Sadir AM, Borca MV (1998). Induction of anti foot and mouth disease virus T and B cell responses in cattle immunized with a peptide representing ten amino acids of VP1. Vaccine.

